# Elevated pCO_2_ Affects Feeding Behavior and Acute Physiological Response of the Brown Crab *Cancer pagurus*

**DOI:** 10.3389/fphys.2018.01164

**Published:** 2018-08-21

**Authors:** Youji Wang, Menghong Hu, Fangli Wu, Daniela Storch, Hans-Otto Pörtner

**Affiliations:** ^1^Key Laboratory of Exploration and Utilization of Aquatic Genetic Resources, Shanghai Ocean University, Ministry of Education, Shanghai, China; ^2^National Demonstration Center for Experimental Fisheries Science Education, Shanghai Ocean University, Shanghai, China; ^3^Department of Integrative Ecophysiology, Alfred-Wegener-Institute, Helmholtz Centre for Polar and Marine Research, Bremerhaven, Germany; ^4^International Research Center for Marine Biosciences at Shanghai Ocean University, Ministry of Science and Technology, Shanghai, China

**Keywords:** pCO_2_, crab, feeding behavior, physiology, specific dynamic action, *Cancer pagurus*

## Abstract

Anthropogenic climate change exposes marine organisms to CO_2_ induced ocean acidification (OA). Marine animals may make physiological and behavioral adaptations to cope with OA. Elevated pCO_2_ may affect metabolism, feeding, and energy partition of marine crabs, and thereby affect their predator-prey dynamics with mussels. Therefore, we examined the effects of simulated future elevated pCO_2_ on feeding behavior and energy metabolism of the brown crab *Cancer pagurus*. Following 54 days of pre-acclimation to control CO_2_ levels (360 μatm) at 11°C, crabs were exposed to consecutively increased oceanic CO_2_ levels (2 weeks for 1200 and 2300 μatm, respectively) and subsequently returned to control CO_2_ level (390 μatm) for 2 weeks in order to study their potential to acclimate elevated pCO_2_ and recovery performance. Standard metabolic rate (SMR), specific dynamic action (SDA) and feeding behavior of the crabs were investigated during each experimental period. Compared to the initial control CO_2_ conditions, the SMRs of CO_2_ exposed crabs were not significantly increased, but increased significantly when the crabs were returned to normal CO_2_ levels. Conversely, SDA was significantly reduced under high CO_2_ and did not return to control levels during recovery. Under high CO_2_, crabs fed on smaller sized mussels than under control CO_2_; food consumption rates were reduced; foraging parameters such as searching time, time to break the prey, eating time, and handling time were all significantly longer than under control CO_2_, and prey profitability was significantly lower than that under control conditions. Again, a two-week recovery period was not sufficient for feeding behavior to return to control values. PCA results revealed a positive relationship between feeding/SDA and pH, but negative relationships between the length of foraging periods and pH. In conclusion, elevated pCO_2_ caused crab metabolic rate to increase at the expense of SDA. Elevated pCO_2_ affected feeding performance negatively and prolonged foraging periods. These results are discussed in the context of how elevated pCO_2_ may impair the competitiveness of brown crabs in benthic communities.

## Introduction

Predation is considered as a significant evolutionary and ecological factor influencing the activity and life style of individuals and the structure and composition of communities (Lima and Dill, 1990). A good knowledge of predation behavior is essential to understand the predator–prey interactions as well as consequences on the food chains (Elner and Hughes, 1978). Crabs have specific feeding habits, and their diets contain many molluscs in coastal and shallow waters. In spite of its economic and ecological importance, the information on the effect of environmental stressors such as seawater pH on crab predation behavior and physiological responses is limited. Therefore, it is of great ecological and economy significance to study the predatory behavior and physiological responses of crabs under global change scenario.

Studies of physiological responses of crabs exposed to elevated pCO_2_ (Pane and Barry, 2007; Spicer et al., 2007; Fehsenfeld et al., 2011) have identified a wide range of responses (Kroeker et al., 2010). High CO_2_ has been reported to negatively impact various physiological processes, including growth (Walther et al., 2010), survival (Melzner et al., 2009), development (Walther et al., 2010; Schiffer et al., 2013), immune response (Meseck et al., 2016), chemoreception (de la Haye et al., 2012), and swimming performance (Dissanayake and Ishimatsu, 2011). The most immediate physiological responses to ocean acidification (OA) in marine crustaceans are best described by acid–base adjustments (Pane and Barry, 2007; Pörtner et al., 2010; Whiteley, 2011). OA causes an increase in CO_2_ in the haemolymph (extracellular compartment), which also enters the intracellular space. A rise in intracellular H^+^ can disrupt key biological processes such as metabolism, protein synthesis, ion-regulation, and cell volume control (Whiteley, 1999). Disruptions to extra- and intracellular acid-base balance have far-reaching consequences by compromising survival and adversely affecting ecologically relevant factors such as metabolism and feeding. High levels of dissolved CO_2_ (hypercapnia) increase the costs associated with acid–base balance (Pörtner et al., 2000), which may result in less energy being available for other energy demanding processes. However, there is currently limited evidence to support this hypothesis in crustaceans.

While the physiological consequences of OA have been studied extensively over the last decade, present knowledge of behavioral changes is limited, especially among invertebrates. OA has been shown to alter behavior in various fish and invertebrates including anti-predator behavior, predator–prey interactions and feeding behavior (Munday et al., 2009, 2014; Ferrari et al., 2011a,b, 2012; Briffa et al., 2012; Nilsson et al., 2012; Schmidt et al., 2017). However, present knowledge is limited across animal groups and little is known about how behavioral effects translate into long-term changes such as reproduction, population dynamics and food chain (Pörtner et al., 2005, 2014; Pörtner, 2008; Gattuso et al., 2011; Wittmann and Pörtner, 2013). General behavioral changes under OA include olfactory discrimination, predator detection, feeding, settlement, lateralization, predatory response (review by Clements and Hunt, 2015). Behavioral changes may also involve changes in the choice of food and the rate of food consumption but also the periods required to search, break, eat, and handle the food (Barbeau and Scheibling, 1994).

So far, seawater acidification has been shown to alter the behavioral responses of crabs and other crustaceans, such as swimming (Dissanayake and Ishimatsu, 2011; Alenius and Munguia, 2012), feeding (Appelhans et al., 2012; Li and Gao, 2012; Saba et al., 2012), assessment and choice behavior (de la Haye et al., 2011), righting response (Zittier et al., 2013), and predator–prey interactions (de la Haye et al., 2012; Landes and Zimmer, 2012; Dodd et al., 2015). Behavioral changes may contribute to large-scale changes in distribution and abundance; however, little information is available on fine-scale feeding responses to OA (de la Haye et al., 2012). Such behavioral changes may be associated with effects on metabolism. The energy cost of maintenance is associated with the rate of standard metabolism. Maintenance cost covered by standard metabolic rate (SMR) includes breathing, blood circulation, ion, and pH regulation, brain and nerve function, and muscular posture. Exercise (e.g., walking or swimming), feeding and digestion demand additional energy. Feeding causes a post-prandial metabolic stimulation called Specific dynamic action (SDA), which depends amongst others on the size of the respective meal. SDA represents the energy demand associated with food processing and is met by an increase in cardiac activity upon food detection in crabs (McGaw and Reiber, 2000). The SDA in crustacea has been measured in response to temperature (Whiteley et al., 2001; Robertson et al., 2002), but whether low pH can affect the SDA response of crabs remains unknown. The mechanistic understanding of changes in feeding behavior, food consumption rates and metabolic consequences of reduced internal pH (acidosis) and increased CO_2_ (hypercapnia) is still incomplete (Appelhans et al., 2012; Landes and Zimmer, 2012). Determining how elevated pCO_2_ might affect the feeding behavior and the associated metabolism of benthic organisms thus appears essential to understand performance changes of individual specimens and, consequently, the impact of these changes on the whole food chain.

The edible crab, *Cancer pagurus*, is one of the most important commercial species in Europe (Johnson et al., 2016), distributed along the Northeast Atlantic coast, from northern Norway to West Africa and present also in the Mediterranean Sea. It lives in a thermal range between about 4 and 16°C depending on season and geographical location (Metzger et al., 2007). The brown crab is an active predator, which feeds on other crustaceans and molluscs (Skajaa et al., 1998). *C. pagurus* has large powerful claws with blunt, broad molars and is a specialist on hard-shelled prey (Yamada and Boulding, 1998). To investigate whether or not exposure to elevated pCO_2_ influences the physiology and behavior of *C. pagurus*, laboratory experiments were conducted to examine the impact of progressive elevated pCO_2_ on oxygen consumption rates including SMR and SDA response, feeding and a set of foraging behaviors. Elevated pCO_2_ may result in decreased feeding rates and energy provision, and thereby reduce its predation on mussels. Identifying the ways how increased CO_2_ may directly affect an individual’s foraging behavior will assist in understanding and anticipating changes in individual survival and fitness, which may have important implications for population and community structure.

## Materials and Methods

### Animal Collection and Maintenance

Wild adult crabs, *C. pagurus* were collected from the subtidal zone around Helgoland (German Bight, North Sea, 54° 11′ N, 7° 53′ E, temperature 5°C, a salinity of 32) and transported to the Alfred-Wegener-Institute in Bremerhaven (Germany) by the German research vessel Uthörn in Feb 2015. Crabs were transported in flow through tanks that were constantly provided with fresh North Sea water. Crabs were maintained in aerated and filtered natural seawater at 8°C and a salinity of 32 in the aquarium facility of the AWI prior to the start of the incubation experiments in Mar 2015, and each crab were fed with two mussels everyday. The water flow rate was 60 l h^-1^, water was exchanged twice a week, and ammonia concentration was kept less than 0.02 mg l^-1^. Only crabs (Body mass = 190 ± 11 g, carapace width = 10.5 ± 0.7 cm, *n* = 12, 6 male, 6 female) in the late inter-molt stage were used in the experiments in order to avoid any potential bias due to behavioral and physiological differences associated with molt stage.

### Experimental Setup

Experiments were designed to test the hypothesis that oxygen consumption rates pre- and post-feeding, feeding and foraging behavior including searching, breaking, eating, and prey handling of *C. pagurus* might be impaired by high CO_2_ levels predicted for the end of this century according to RCP 8.5 (Caldeira, 2005; Gattuso et al., 2015). The CO_2_ incubation systems were set up in a temperature-controlled room (10°C) using four reservoirs (450 l) and two header tanks (210 l) to provide experimental treatment conditions according to oceanic CO_2_ levels. There were two identical and independent experimental systems, each consisting of a header tank (210 l) that was connected with six individual aquaria of 20 l that hosted one crab each. From there, water overflowed (flow rate 60 l h^-1^) into a water-collecting tank (200 l) underneath and was pumped into the water-providing reservoir (450 l, there were two reservoirs for each system), which was connected with the header tank (210 l). Water was circulated through the reservoir, the header tank and the experimental tanks. Both the reservoir and the header tank were continuously bubbled with the respective air–CO_2_ mixture, providing stable CO_2_ conditions (**Table 1**). Water was exchanged twice a week by disconnecting the reservoir from the system, and connecting to another reservoir with fresh seawater set to the respective experimental CO_2_ condition. The experimental CO_2_ concentrations were achieved by continuously bubbling the water with an air–CO_2_ mixture produced by a MKS mass flow controller (MKS Instruments Deutschland GmbH, München). During experiments, each tank was covered with transparent plastic sheeting to minimize external disturbance, and all experiments were carried out in constant dimmed light of fluorescent LED. The CO_2_ levels were gradually changed from a baseline level of pH (pH8.1) to the required test treatments (pH 7.7 and 7.4) within 12 h. Water temperature and salinity were monitored daily by a salinometer (LF197, WTW, Weilheim, Germany). The partial pressure of CO_2_ (pCO_2_) was measured once a week in all tanks (experimental, header, and reservoirs tanks) by using a combined carbon dioxide probe (CARBOCAP GMP343, Vaisala, Helsinki, Finland) and carbon dioxide meter (CARBOCAP GM70, Vaisala). pH_(NBS)_ was measured by a pH meter (pH3310, WTW, Weilheim) calibrated with NIST buffers (pH 6.865 and 9.180) at the incubation room temperature. Other water parameters, including total alkalinity (TA), dissolved inorganic carbon (DIC), saturation states of calcite (Ωca) and aragonite (Ωar) were calculated based on the measured parameters (pCO_2_ and pH) using the CO_2_SYS program (Pierrot et al., 2006) after equilibrium constants of Mehrbach et al. (1973) for the CO_2_/bicarbonate/carbonate system, as refitted by Dickson and Millero (1987) and used for KSO_4_ as provided by Dickson (1990) (**Table 1**).

**Table 1:** Carbonate chemistry of seawater during the incubation of brown crabs, *C. pagurus* at different CO_2_ concentrations (*n* = 12).

Treatments	S	T	pH_NBS_	TA	DIC	pCO_2_	Ωcal	Ωara
pH		(°C)		(μmol Kg^-1^)	(μmol Kg^-1^)	(μatm)		
8.10	32.1 ± 0.07	10.77 ± 0.09	8.18 ± 0.004	2483 ± 42	2267 ± 40	363 ± 10	3.93 ± 0.05	2.49 ± 0.03
7.70	31.5 ± 0.02	10.86 ± 0.07	7.69 ± 0.007	2383 ± 28	2357 ± 29	1232 ± 28	1.34 ± 0.02	0.84 ± 0.02
7.40	31.7 ± 0.13	10.77 ± 0.10	7.41 ± 0.013	2323 ± 73	2380 ± 71	2317 ± 48	0.71 ± 0.04	0.45 ± 0.03
8.10 (recovery)	32.4 ± 0.12	10.68 ± 0.06	8.17 ± 0.003	2441 ± 28	2231 ± 26	364 ± 4	3.80 ± 0.05	2.40 ± 0.03

### Incubations

Before separating the crabs in single aquarium of the experimental setup, crabs were cleaned and freed from epibionts. Animals were exposed at 10°C and a salinity of 32 in a temperature controlled room. Crabs were randomly placed in 12 tanks (20 l) and held for 54 days under control pH conditions before low pH exposure to ensure acclimation to the experimental conditions, and the physiological activities of crabs were stable during this period. During the acclimation time, each crab was fed with frozen soft tissues of blue mussel *Mytilus edulis* (ca. 6 g) every day and experimental tanks were cleaned from feces on a daily basis. After the incubation at control pH (pH 8.18 ± 0.004), the crabs were successively exposed for 2 weeks at 1232 μatm CO_2_ (pH 7.69 ± 0.007) and for 2 weeks at 2317 μatm CO_2_ (pH 7.41 ± 0.013), respectively. After the incubation at the two increased CO_2_ levels for 4 weeks, animals were returned to control conditions for 2 weeks to detect their potential for recovery. pH values were 8.18 ± 0.004 (control), pH 7.69 ± 0.007, pH 7.41 ± 0.013, and pH 8.17 ± 0.003 (recovery). The animals were starved for 2 days prior to metabolic experiments (Curtis and McGaw, 2011). This starvation period ensured that there was no food residue in the gut, but avoided the effects of long-term starvation. At each CO_2_ condition, all crabs were tested for metabolic activities and feeding behaviors within a two-week period in an order of SMR (7th day), SDA response (8th day), prey size selection (10th day), feeding rate (12th day), and foraging behavior (14th day). The crabs were exposed to elevated pCO_2_ for 4 weeks in total.

### Oxygen Consumption Rates and SDA

Oxygen consumption rates (MO_2_) of crabs were measured in an intermittent flow-through respiration chamber (V_chamber_ = 3750 ml), which was submerged in a tank filled with 60 l seawater set to the respective CO_2_ condition by continuously equilibrating the seawater with the relevant air-CO_2_ mixture. This system was equipped with two pumps, the first pump continually flushed fresh seawater through the chamber while it was open, and the second pump recirculated the water around the chamber when it was closed for measurements, eliminating oxygen gradients in the chamber. The tank was covered with black plastic films to prevent external disturbance. The periodic activation of the flush pump connected with the respirometer was controlled by a mechanic clock timer. This flushing process was operated for 15 min once every hour with a flow rate of 300 l h^-1^, and then the oxygen consumption rate was recorded for 45 min at a flow rate of 490 l h^-1^. The percentage of oxygen saturation was measured continuously every 30 s by an optical oxygen meter equipped with a fiberglass optode (FIBOX 3; PreSens, Regensburg, Germany), and recorded by the relevant software (PSt3, version 7.01; PreSens, Regensburg). According to preliminary experiments, the crabs were held in the respiration chamber for 24 h, which was sufficient to identify stable metabolic rates and SDA response. Throughout the 24 h periods, the lowest 10% of measured metabolic rates, stable for at least 40 min were classified as SMR. For SDA measurements, the crabs were fed a meal of mussel tissues, all had finished feeding by the time the first postprandial oxygen consumption reading (0.5 h) was completed. Every time, four crabs were measured at the same time by using four respirometry systems. In this way, SDA response was measured 48 h after SMR measurement for the same crab, which allowed the crab recover from the chamber. During the measurement, the 10% highest metabolic rates were classified as peak metabolic rate post feeding (Peak MO_2_), and the SDA of each animal was calculated from the difference between peak MO_2_ and SMR and standardized to kJ using the conversion factor of 1 mg O_2_ = 14 J (Secor, 2009). Oxygen consumption of crabs (MO_2_) was expressed as nmol O_2_ min^-1^ g^-1^ fresh weight. Based on the measured percent values of oxygen saturation, the oxygen consumption rate was calculated.

The oxygen concentration (c_O2_) of 100% air saturated seawater was calculated as

(1) $$c_{O2}=\alpha_{O2}\cdot [(P_{air}-P_{wv})\cdot 0.2095]$$

where α_O2_ is the Bunsen solubility coefficient of oxygen (μmol l^-1^ torr^-1^) at a given temperature and salinity, P_air_ is the pressure of air, P_wv_ is the vapor pressure of water and 0.2095 represents the oxygen percentage in air (Boutilier et al., 1984). The oxygen concentration is given in μM. The absolute amount of oxygen of the respiration chamber (c_O2ch_) was calculated accordingly:

(2) $$\left(c_{O2ch})=c_{O2}\cdot (V_{chamber}-V_{ind.}\right)$$

where V_chamber_ is the volume of the chamber (l) and V_ind._ corresponds to the volume of the measured animal (l) which was estimated by the fresh weight (g) of the crab. Subsequently, the decrease of oxygen in the chamber over time was used to calculate the mass-specific oxygen consumption rate (*M*O_2_) of each individual:

(3) $$MO_{2}=c_{O2ch}\Delta c_{rel}/(\Delta t\cdot w_{f}\cdot 100)$$

where w_f_ is the fresh weight of the crab. With the fresh weight of the crab (wf) and the absolute amount of oxygen at 100% air-saturation, the recorded change of saturation over time (Δcrel. Δt-1) can be used to calculate the mass-specific oxygen consumption rate MO_2_ of each crab. The change in oxygen saturation (the mean descending slope of the saturation) was averaged over the time period of 45 min when the chamber was closed. The mean slopes were derived by a LabChart Reader (v8.0.5; ADInstruments, Oxford, United Kingdom). With the selected setup, one value for MO_2_ was taken as a representative mean for 1 h of measurement.

For SDA measurements, the crabs were fed soft tissue (ca. 6 g) of the mussel *M. edulis* (without shell) for 1 h outside of the chamber. Subsequently, crabs were quickly placed in the respiration chamber and oxygen consumption rates were measured as described above. Oxygen consumption was recorded until it returned to pre-feeding levels within 24 h. Besides maximal oxygen consumption after the meal (peak MO_2_), the following parameters were calculated for each experiment: (a) the time to reach the peak oxygen consumption following feeding, (b) the scope of the SDA–peak MO_2_ divided by SMR, (c) the duration of the SDA response – until oxygen returned to pre-feeding levels, and (d) the SDA of each animal in kJ (as a function of individual mass). SDA co-efficient expresses the proportion of the digested energy used for the digestive process and is calculated by dividing the SDA by the energy of the meal (kJ). The energy equivalent of the meal was calculated from the amount of food ingested (g) multiplied by the calories per g of mussel (22j mg^-1^, Bayne and Worrall, 1980).

### Prey Size Selection

Prey size selection experiments were carried out by presenting individual crabs with live *M. edulis* ranging from 10 to 70 mm shell length. Each crab was simultaneously offered five prey items of each size class (size classes were defined according to the following shell length: 10–20, 21–30, 31–40, 41–50, 51–60, 61–70 mm). Prey items were scattered randomly over the floor of the aquaria. Any item consumed by the crab was recorded over a period of 24 h. The number of consumed mussels of each size class was counted and expressed as “number of mussels eaten day^-1^ (*n* = 12 crabs). Comparisons of the size ranges of prey preferred by crabs in all treatments were made on the basis of shell length. All following experiments on feeding rate and foraging behavior were carried out with the preferred size class “41–50 mm” according to the control treatment (pH 8.1).

### Feeding Rate

After the prey size selection experiment, crabs were fed with mussels with pre-determined size, i.e., shell length 41–50 mm. According to the prey size selection experiment, the crabs were fed with enough mussels for 1 day, and the numbers of mussels consumed by each crab were recorded. Consumption rate was calculated as the number of mussels eaten day^-1^ for each crab (*n* = 12).

### Foraging Behavior and Prey Profitability

Each tank was continuously monitored using a video camera (Coomatec DVRCam C802H) connected to a computer. The camera was positioned about 1.2 m above the tank in order to monitor the whole bottom area and to follow the movements of crabs. The crabs were starved for 2 days and the trial was initiated when one mussel was lowered into the water in the field of crab vision. Experiments to compare the foraging behavior and profitability of consumed mussels were carried out by offering crabs a prey item (shell length 40 mm). Prey profitability is the yield of flesh per unit of handling time and calculated by dividing the estimated flesh weight of the prey by the handling time. The following parameters were measured: (1) Searching time (St), defined as the time from when the mussel was put into the tank until the crab grabbed it with its claws; (2) Breaking time (Bt) defined as the time from the crab’s first physical contact with the prey, through the period of shell crushing, to the first bite of exposed mussel tissue: (3) Eating time (Et), time from the first bite to the abandonment of the open shell, including time spent re-manipulating and re-breaking the shell so as to extract the mussel tissue; (4) Handling time (Ht), sum of both breaking time and eating time were recorded; (5) Prey profitability (i.e., prey size yielding the most flesh per unit handling time), was calculated by dividing the estimated dry weight of the mussel by the handling time (g s^-1^).

Dry flesh weight of *M. edulis* prey items was calculated on the basis of the allometric equations reported by Mascaró and Seed (2001b):

(4) log(W)=−4.94+2.69log(SL)

where W is dry flesh weight (g) and SL is the maximum shell length (mm).

### Statistical Analysis

The SPSS 17.0 software package was used for statistical analyses. Both the normal distribution (Shapiro–Wilk’s test) and the homogeneity of the variance (Levene’s test) were assessed. One way ANOVA followed by Tukey’s multiple comparison was performed to compare the difference between control and high CO_2_ conditions as well as the recovery condition for physiological and behavioral responses. Relationships between any two of SMR, peak MO_2_ and SDA as well as between any one of SMR, peak MO_2_ and SDA with each feeding/foraging parameter (i.e., feeding rate, searching time, breaking time, eating time, handling time, prey profitability) were tested with linear regression analysis. Principal component analysis (PCA) was carried out by using XLSTAT^®^2014 and used to find the main cause of induced responses as well as the relationships among these parameters. A biplot was graphed with both the measured parameters and the observations. All data were expressed as means ± standard error (SE).

## Results

### Monitoring of pH in the Experimental Tanks

The differences in pH and carbonate chemistry of seawater between different CO_2_ exposure periods were compiled in **Table 1**. Temperature and salinity values of the exposure tanks were consistently maintained at 11°C and a salinity of 32, respectively. TA ranged from 2212 to 2690 μmol kg^-1^. The CO_2_ system was generally effective in maintaining three clearly distinct levels of pH during acclimation, experimental, and recovery periods.

### Oxygen Consumption Rates and SDA

**FIGURE 1:**
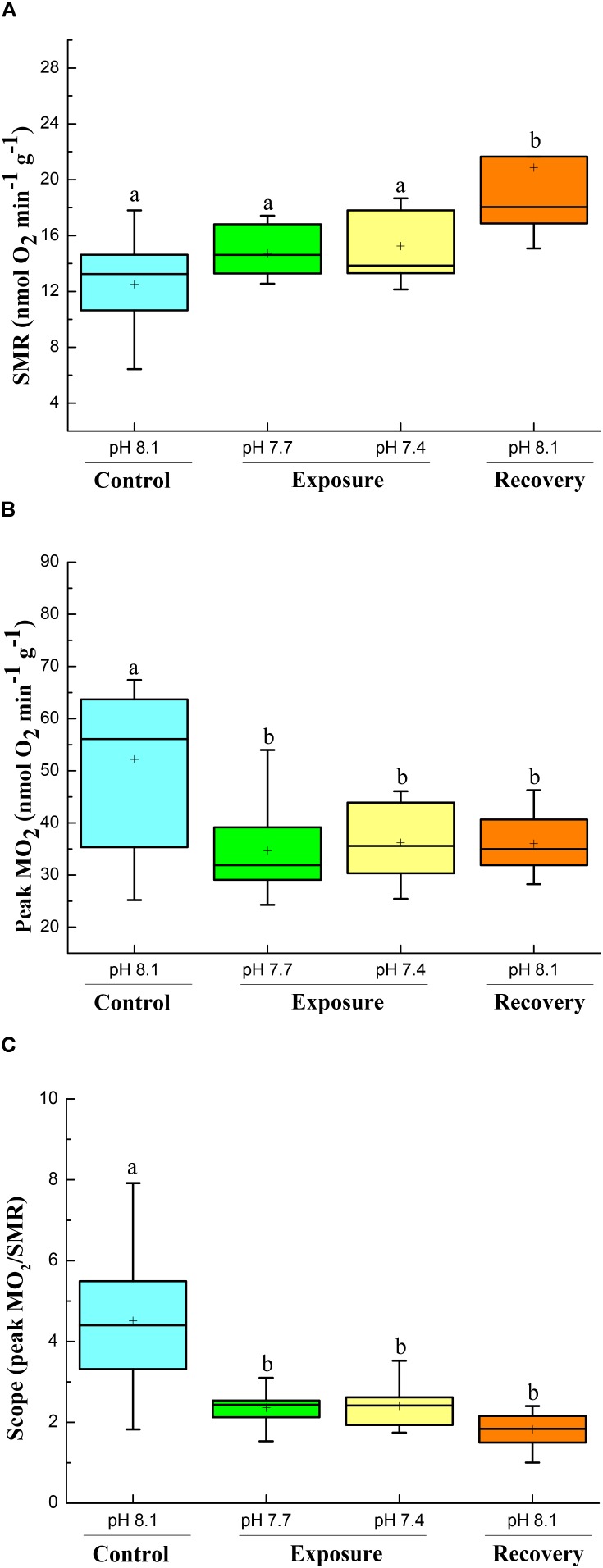
SMR **(A)**, Peak MO_2_ **(B)**, and scope **(C)** of *C. pagurus* at varying pH conditions (pH 8.1 control), pH 7.7, pH 7.4, and pH 8.1 (recovery). Data were analyzed using one-way ANOVA. Significance was reported at the *P* < 0.05 levels, Different letters indicate that the value was significantly different from the control pH 8.1.

Based on one way ANOVA results, all parameters were significantly affected by CO_2_ exposure, and did not recover to control levels within 2 weeks when crabs were returned to control conditions (*P* < 0.05). SMR was not significantly increased when crabs were exposed to low pH conditions compared to their initial control period, but increased significantly when they were returned to control conditions (pH 8.17) during recovery (**Figure 1A**). However, the peak metabolic rate (Peak MO_2_) and scope (peak MO_2_/SMR) after feeding showed reverse patterns (**Figures 1B,C**); they decreased significantly when crabs were exposed to low pH conditions, and did not recover when crabs were returned to pH 8.17 (recovery) (**Figures 1B,C**).

**FIGURE 2:**
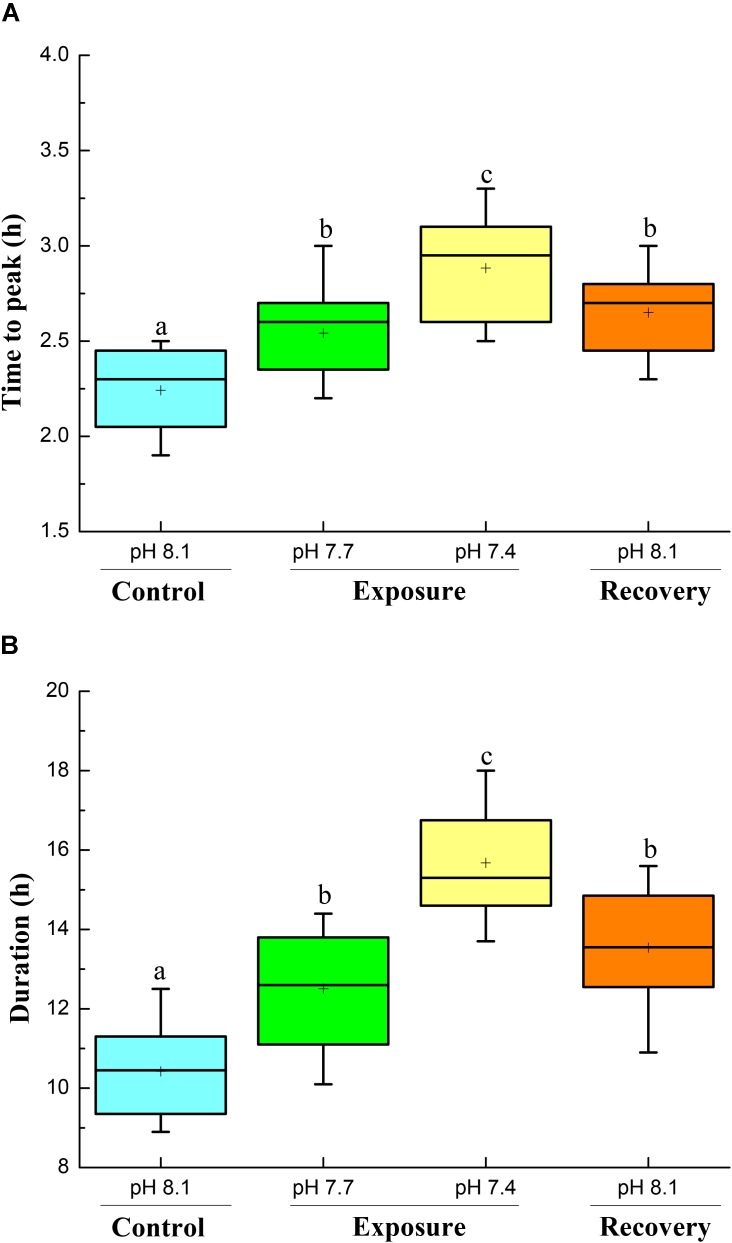
Time to peak **(A)** and SDA duration **(B)** of *C. pagurus* at varying pH conditions (pH 8.1 control), pH 7.7, pH 7.4, and pH 8.1 (recovery). Data were analyzed using one-way ANOVA. Significance was reported at the *P* < 0.05 levels, Different letters indicate that the value was significantly different from the control pH 8.1.

**FIGURE 3:**
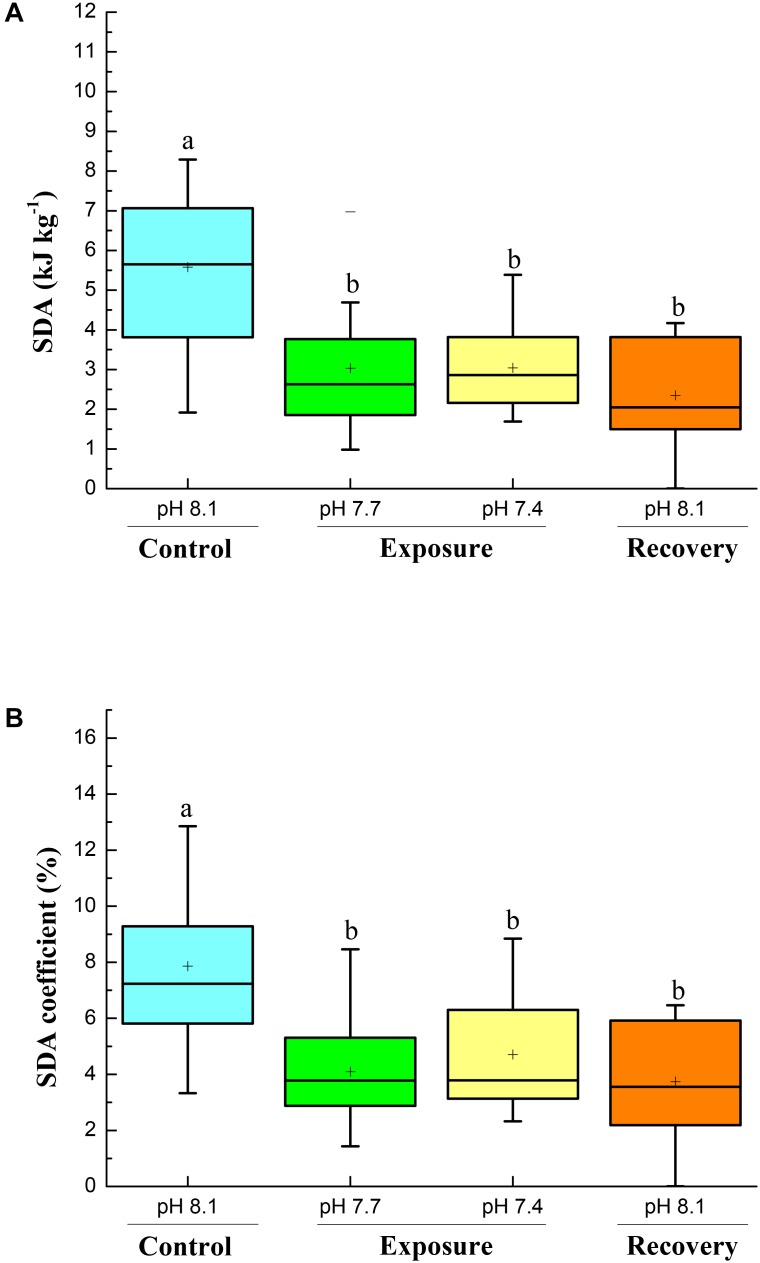
SDA (kJ kg^-1^, **A**) and SDA coefficient **(B)** of *C. pagurus* at varying pH conditions (pH 8.1 control), pH 7.7, pH 7.4, and pH 8.1 (recovery). Data were analyzed using one-way ANOVA. Significance was reported at the *P* < 0.05 levels, Different letters denote that the value was significantly different from the control pH 8.1.

Specific dynamic action characteristics are shown in **Figures 2**, **3**. The time to peak post-prandial MO_2_ increased significantly with pH reduction and reached the highest value at pH 7.4 (**Figure 2A**); however, after 2 weeks of recovery at pH 8.17 the time to peak MO_2_ decreased but was still higher compared to the initial control (**Figure 2A**). The duration of the SDA was prolonged compared to control conditions. The SDA duration time significantly increased with pH reduction with the highest value at pH 7.4, and did not return to control pH levels after 2 weeks of recovery (**Figure 2B**). Low pH exposure significantly reduced SDA responses and SDA coefficient, and consecutive short-term acclimation under control conditions did again not lead the crabs to recover to their initial state (**Figures 3A,B**).

### Prey Size Selection

**FIGURE 4:**
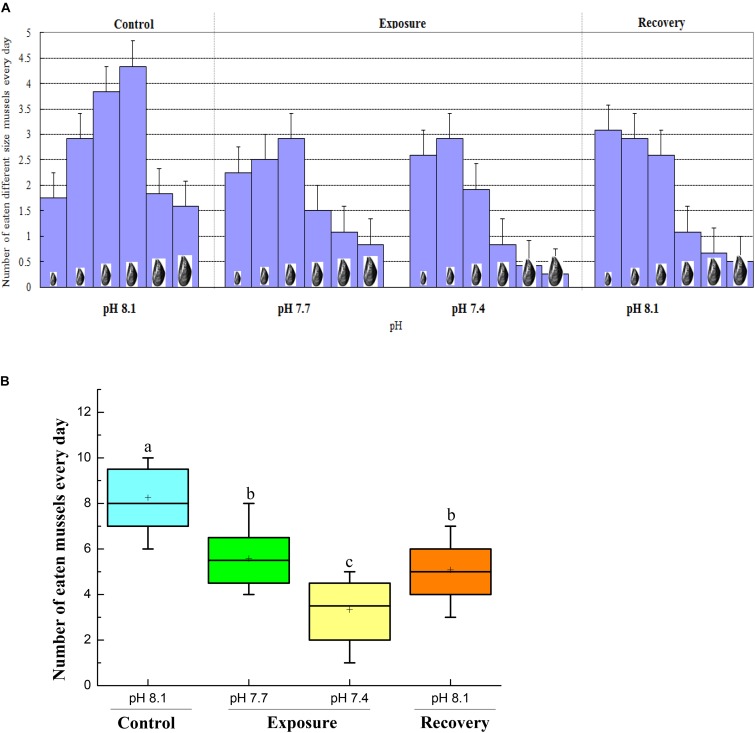
Prey-size selection **(A)** and feeding rate of same-sized mussels (41–50 mm) **(B)** of *C. pagurus* at different pH conditions. Values are mean consumption rates ± SE (*n* = 12) expressed as number of eaten *Mytilus edulis* crab^-1^ d^-1^. Increased triangles denote increased mussel size, from small to big, shell length ranged from 10–20, 21–30, 31–40, 41–50, 51–60, 61–70 mm. Different letters indicate significant differences compared to initial control pH conditions (*P* < 0.05).

When the crabs were cultured under control conditions, they preferred to eat mussels of medium size (30–50 mm shell length). However, under low pH conditions, the crabs tended to prey on mussels of smaller size (10–30 mm shell length for pH 7.7, 10–20 mm shell length for pH 7.4). When the crabs were returned to pH 8.17 for recovery, they continued to select the smallest mussels (**Figure 4A**).

### Feeding Rate

The feeding rate of crabs decreased significantly when they were exposed to pH 7.7, and a further significant decrease was observed when they were exposed to pH 7.4. Although the feeding rate increased after the crabs were returned to normal conditions, it remained significantly lower than that under initial control conditions (**Figure 4B**).

### Foraging Behavior

**FIGURE 5:**
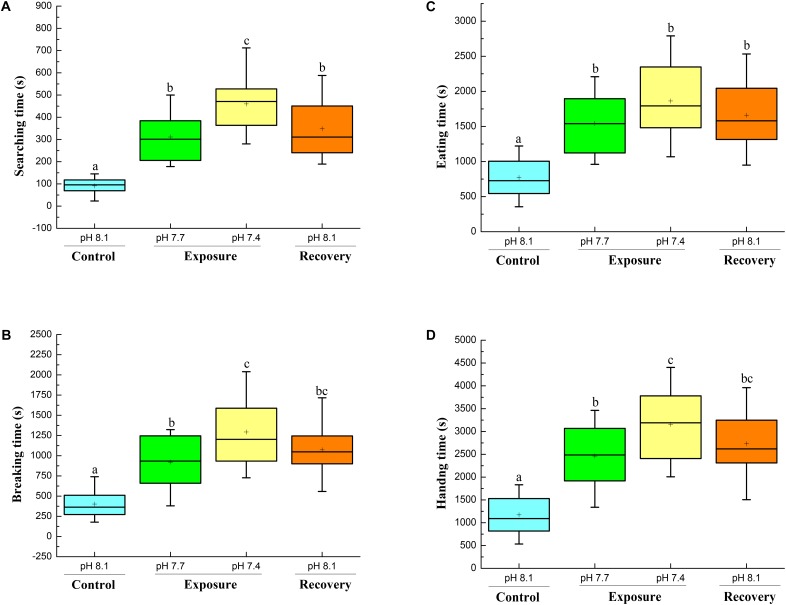
Foraging behavior of *C. pagurus* at different pH conditions: Searching time **(A)**, Breaking time **(B)**, Eating time **(C)**, and Handling time **(D)** of *C. pagurus* at different pH conditions following four successive treatments. Values are means ± SE. Different letters indicate significant differences compared to initial control pH conditions (*P* < 0.05).

The searching time significantly increased when the crabs were exposed to pH 7.7, and a further significant increment was found when the crabs were exposed to pH 7.4. When the crabs were returned to control conditions (pH 8.17 recovery), the searching time decreased, but remained significantly higher compared to initial control conditions (**Figure 5A**).

The mean breaking time more than doubled when the crabs were exposed to pH 7.7 in comparison to control pH 8.18 and tripled when crabs were exposed to pH 7.4. When the crabs were back to control conditions, the breaking time decreased, but was still significantly higher than under initial control conditions (**Figure 5B**).

The eating time significantly increased when the crabs were exposed to pH 7.7 and pH 7.4. When the crabs were back to normal conditions, the eating time decreased a little, but was still significantly longer than the initial one (**Figure 5C**).

The handling time increased significantly when crabs were exposed to pH 7.7, and a further significant increment was found when the crabs were exposed to pH 7.4. When the crabs were back to normal conditions, the handling time decreased, but was still significantly longer than that under initial control conditions (**Figure 5D**).

### Prey Profitability

**FIGURE 6:**
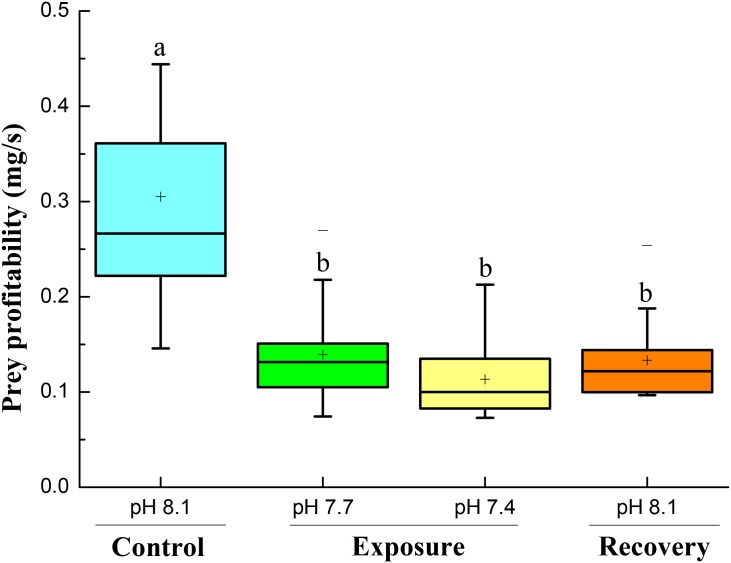
Prey profitability of crabs at different pH conditions following four successive treatments. Values are means ± SE expressed as mg dry weight handling time^-1^ crab^-1^. Different letters indicate significant differences between pH conditions (*P* < 0.05).

Prey profitability decreased significantly when the crabs were exposed to pH 7.7 and pH 7.4. When the crabs were back to normal conditions, prey profitability increased a little, but remained significantly lower than under initial control conditions (**Figure 6**).

### Correlations Between Parameters and PCA

**FIGURE 7:**
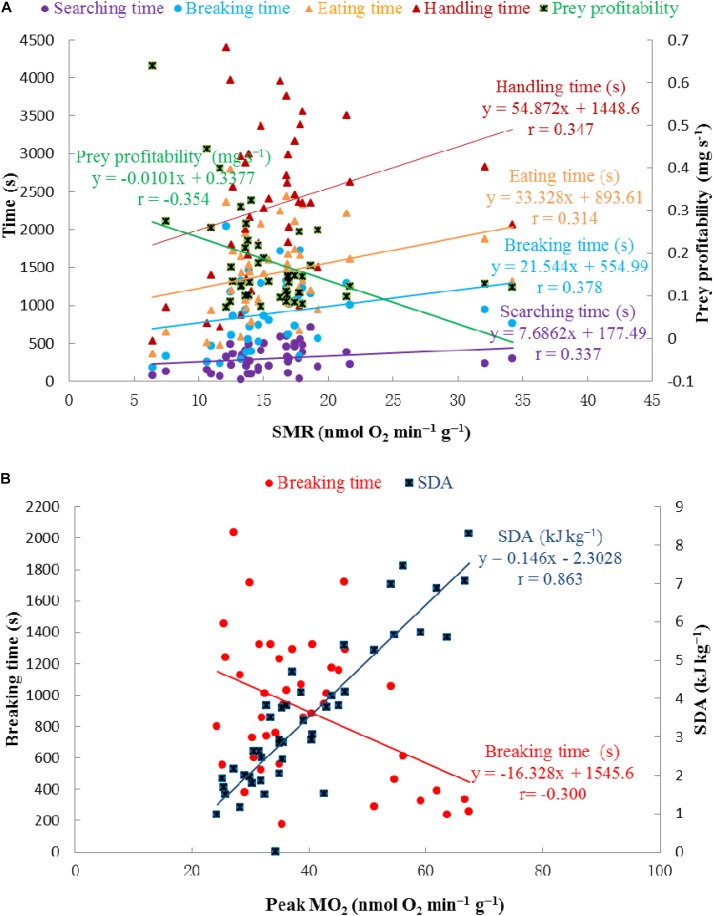
Correlations between SMR (nmol O_2_ min^-1^ g^-1^) and searching time, breaking time, eating time, handling time, prey profitability, respectively **(A)** and correlations between peak MO_2_ (nmol O_2_ min^-1^ g^-1^) and SDA (kJ kg^-1^), breaking time, respectively **(B)**.

Significant correlations were observed between SMR and each item of foraging behavior, as well as prey profitability, and between peak MO_2_ and SDA, as well as breaking time (**Table 2** and **Figure 7**).

**Table 2:** Relationships of physiological activities [SMR (nmol O_2_ min^-1^ g^-1^), Peak MO_2_ (nmol O_2_ min^-1^ g^-1^), and SDA (kJ kg^-1^)] and foraging/feeding behaviors (feeding rate, searching time, breaking time, eating time, handling time, and prey profitability) were tested by means of Spearman correlation.

			SMR	Peak MO_2_	SDA	Feeding rate	Searching time	Breaking time	Eating time	Handling time	Prey profitability
Spearman’s rho	SMR	Correlation coefficient	1.000	-0.055	-0.243	-0.282	0.337*	0.378*	0.314*	0.347*	-0.354*
		Sig.	–	0.724	0.112	0.063	0.025	0.011	0.038	0.021	0.018
		N	44	44	44	44	44	44	44	44	44
	Peak MO_2_	Correlation coefficient	–0.055	1.000	0.863**	0.269	-0.252	-0.300*	-0.294	-0.291	0.287
		Sig.	0.724	–	0.000	0.078	0.099	0.048	0.053	0.055	0.059
		N	44	44	44	44	44	44	44	44	44
	SDA	Correlation coefficient	–0.243	0.863**	1.000	0.280	-0.200	-0.235	-0.273	-0.256	0.249
		Sig.	0.112	0.000		0.066	0.192	0.125	0.073	0.094	0.103
		N	44	44	44	44	44	44	44	44	44

*Correlations between SMR and searching time, breaking time, eating time, handling time, prey profitability, respectively, and correlations between peak MO_2_ and SDA, breaking time, respectively, were tested with linear regression analysis. ^∗^Correlation is significant at the 0.05 level (2-tailed). ^∗∗^Correlation is significant at the 0.01 level (2-tailed)*.

**FIGURE 8:**
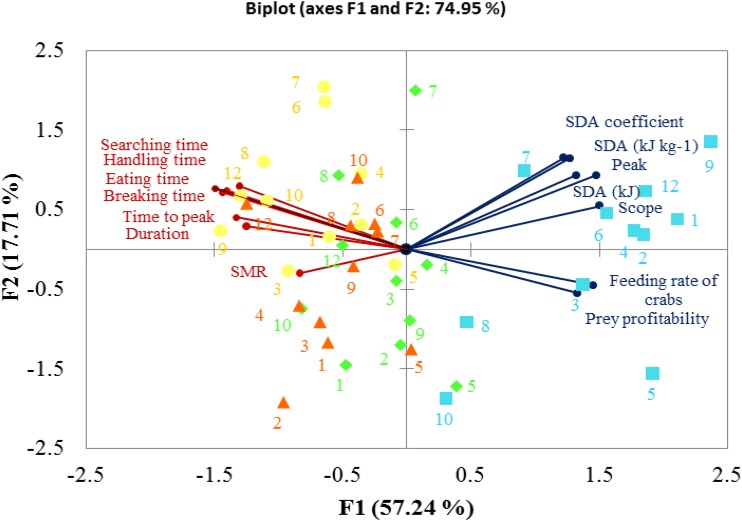
Biplot originating from a principal component analysis integrating all variables measured in *C. pagurus* following four progressive treatments (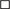
–initial pH 81, 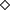
–pH 7.7, 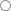
–7.4, 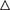
–recovery pH 7.7). Both the loadings of the variables (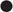) and the scores of the experimental conditions are shown.

Principal component analysis revealed that 74.95% of overall variance was explained by two principal components during the whole experiment. PC1 expressed 57.24% of overall variance, showing the most significant result when comparing the initial control pH conditions and subsequent CO_2_ treatments. The large SDA responses were grouped together, showing negative correlations with SMRs, foraging behaviors were also grouped and their time duration correlated negatively with feeding rate and prey profitability (**Figure 8**).

## Discussion

### Standard Metabolic Rate

Respiration is a commonly used metric of stress in marine organisms. In our experiment, the SMR was 10–20 μmol O_2_ kg^-1^ min^-1^, which was similar to previous results for *C. pagurus* (8–19, Naylor et al., 1997). Increased metabolic rates under elevated pCO_2_ conditions were observed in crab (Schiffer et al., 2013) and other invertebrates (Lannig et al., 2010; Thomsen and Melzner, 2010; Parker et al., 2012), implying these species have an ability to at least partially compensate for the increased energy costs of acidosis (Gibson et al., 2012). However, some opposite studies have shown that oxygen consumption in *Metacarcinus magister* acclimated to high pCO_2_ for 7 days was significantly decreased (Hans et al., 2014), indicating that *M. magister* undergoes partial metabolic depression under elevated pCO_2_. Metabolic depression as a response to elevated environmental pCO_2_ and the following respiratory acidosis are usually associated with an uncompensated pHe shift (Pörtner et al., 2004). In our study, metabolic rates increased under elevated pCO_2_ conditions, thus stressful conditions appear to negatively impact crabs by increasing metabolic rates, which would decrease energy available for other aspects, such as feeding behavior. The metabolic costs of survival in altered environments can result in reduced energy available for other behaviors, such as foraging and feeding (Briffa et al., 2012). For instance, in our study, increased metabolic costs caused by elevated pCO_2_ may cause *C. pagurus* to reduce feeding rate.

In the present study, the SMR of CO_2_-exposed animals was higher than that of the normocapnic controls, indicating that hypercapnia resulted in elevated energy demand. Shifts in the cellular energy budget toward higher acid-base and ion-regulatory demands under elevated pCO_2_ and a concomitant reduction of, e.g., protein biosynthesis may have been involved (Deigweiher et al., 2010). These shifts, in turn, may lead to reductions of energetically more costly processes such as feeding and digestion (McGaw and Twitchit, 2012), in which SDA responses were impaired in the present study. It is known that elevated seawater pCO_2_ leads to decreasing extracellular pH in crustaceans (Pane and Barry, 2007). There is also evidence that an uncompensated drop in extracellular pH has a depressing effect on aerobic energy metabolism of some tissues like muscle (Reipschläger and Pörtner, 1996) through effects on the mode and rate of proton equivalent ion exchange and a decrease in protein synthesis (Pörtner et al., 2000). This may result in a decreased whole animal oxygen consumption (Pörtner et al., 1998; Michaelidis et al., 2005) or, if compensated for by the rise in energy demanding processes (e.g., calcification), an increase in whole organism oxygen demand (Thomsen and Melzner, 2010; Stumpp et al., 2011). In the study of Wood et al. (2008), respiration rates of the brittlestar *Amphiura filiformis* exposed to hypercapnia were increased. The potential impact of elevated pCO_2_ on aerobic scope and metabolic rates have been assessed in a number of fish species with the general hypothesis that the cost of coping with elevated pCO_2_ (acid–base and osmoregulation as well as cardiorespiratory adjustments) would increase SMR and/or cause a shift in energy budget and reduce aerobic scope and finally fitness (Kreiss et al., 2015).

### Specific Dynamics Action

Specific dynamic action reflects the energy involved in feeding activity, mechanical breakdown of food, intracellular and extracellular digestion and the subsequent protein and lipid synthesis (McGaw and Reiber, 2000; Whiteley et al., 2001; Mente et al., 2003; McCue, 2006; Secor, 2009; McGaw and Whiteley, 2012). In temperate crustaceans, SDA is apparent as a two to fourfold increase in oxygen uptake, which usually reaches peak values within 4 h of feeding and may remain elevated for over 48 h (McGaw and Reiber, 2000; Robertson et al., 2002; Mente et al., 2003; McGaw, 2006; McGaw and Curtis, 2013), and this is also the case in the present study.

Previously, many studies of SDA were based on the food types (Secor, 2009; McGaw and Curtis, 2013), while less information is available on the feedbacks by environmental impacts (McGaw and Whiteley, 2012), for example, elevated pCO_2_. The effect of low pH stress on the SDA of crustaceans was observed as decreases in peak MO_2_, scope and SDA and increases in duration and peak size of the SDA response in our study. The reduction in factorial scope at low pH might be due to the fact that the animals are closer to their upper limit of acid-base regulation and are unable to increase their metabolic rate in proportion to meal size. This reduced scope for digestion (SDA factorial rise) suggests an impairment of the mechanisms associated with feeding and digestion, such as the depression of intracellular protein synthesis that accounts for a large proportion of the increase in postprandial metabolic rate (Mente et al., 2003). The total SDA (energy expended in kJ) is a function of both its scope and its duration. When exposed to low pH following feeding, besides digestion, *C. pagurus* also spends energy on acid-base regulation, and thus reduces the SDA response. An alternative explanation for the observed decrease in oxygen uptake (SDA) for *C. pagurus* is that this species simply lacks the resources to divert to digestion during CO_2_ exposure, which has been discussed for *Cancer magister* exposed to low salinity (Curtis and McGaw, 2010).

As CO_2_ reduced the overall SDA response, other physiological and behavioral aspects may also be influenced, i.e., foraging and reproduction may be reduced. For example, hermit crabs *Pagurus bernhardus* exposed to reduced pH showed declines in the locomotory activity (de la Haye et al., 2012) and the abilities of resource assessment and decision-making (de la Haye et al., 2011). Changes in chemo-responsiveness might simply reflect reduced activity levels, or reduced responsiveness to chemical cues, occurring as a result of the elevated metabolic load of maintaining acid–base balance under conditions of low pH (Pörtner et al., 2004). It is possible that reduced pH could cause physical damage to sensory organs, as calcified animals may experience dissolution of their exoskeletons under such conditions (Spicer et al., 2007; Hall Spencer et al., 2008). In addition, physicochemical stress is likely to affect energetically demanding activities, such as foraging, as seen in this study. The correlation between SMR and foraging/feeding times (**Figure 7A**), indicates that physiological effects may drive behavioral changes in crabs when they are exposed to high pCO_2_ conditions.

### Prey-Size Selection

Prey size selection has been reported in numerous studies of crab feeding behaviors (e.g., Elner and Hughes, 1978; Arnold, 1984; Juanes, 1992; Hughes and Seed, 1995; Mascaró and Seed, 2000a,b, 2001a,b), and considered within the context of optimal foraging theory, whereby a predator selects its diet to maximize net energy intake per unit of handling time (Elner and Hughes, 1978; Hughes, 1980; Hughes and Seed, 1981). Profitability of *M. edulis* as a prey item has been determined in this study, and the mean prey profitability decreased with increased pCO_2_, mainly because of increases in breaking and eating time. Theoretically, crabs select the most profitable of alternative prey sizes (i.e., those yielding the most flesh per unit handling time), and take less profitable prey only when opportunity for choice becomes limited (Elner and Hughes, 1978; Smallegange and van der Meer, 2003; Smallegange et al., 2008). Under optimal diet conditions (Hughes, 1980), preference for prey is often a compromise between the energy demand for prey handling and energy gain from prey consumption. In the present study, under normal pH conditions, preying on large mussel required longer handling times and presumably more energy demand, but enhanced energy intake may compensate for the energy demand and thus explain the consumption of larger preys. Brown crabs exposed to high pCO_2_ selected smaller mussels because small mussels are easier to crush and the crab needs less energy to consume the smaller mussels. During 2 weeks of recovery, crabs still selected smaller mussels, indicating that they had not fully recovered. This is further emphasized by extremely high SMR during recovery compared to all other treatments likely due to increased acid-base regulation to readjust homeostasis. Therefore, less energy was available for feeding and digestion, also mirrored by the low scope of peak MO_2_ and SDA.

Preferring medium-sized prey (mussels with medium shell lengths) also appears to be the mechanism underlying prey selection by *C. maenas* (Elner and Hughes, 1978; Burch and Seed, 2000). Under normal pH conditions *C. pagurus* also consumed more medium sized *M. edulis* (4–5 cm) than small and big mussels. Many factors play a role in prey size selection for crabs, including the size relationship between crab and prey, degree of satiation, claw gape, claw strength, dentition, and total prey-handling time (Yamada and Boulding, 1998). In our study, all crabs were subject to the same degree of satiation and fed with same mussels. Thus, crabs exposed to elevated pCO_2_ preferred smaller mussels, probably due to the reduced claw strength and increased handling time, reducing the capacity to crash shell. In the shore crab *C. maenas*, acidification negatively affected the closer-muscle length of the crusher chela and correspondingly the claw-strength (Landes and Zimmer, 2012). Overall, it may be more difficult for *C. pagurus* exposed to elevated pCO_2_ to crush mussel shells, visible as an increase in prey handling time and a shift to smaller prey.

### Food Consumption and Foraging Behavior

High pCO_2_ (3500 μatm) has been shown to reduce feeding performance in various invertebrates (Siikavuopio et al., 2007; Appelhans et al., 2012; Stumpp et al., 2013). Similar to these studies, food consumption was affected by elevated pCO_2_ in *C. pagurus*. Reduced feeding rates may result from a number of physiological processes, but underlying mechanisms need to be further investigated. One explanation based on the present study is that the foraging activity of crab was impaired at higher pCO_2_ levels as indicated by Dodd et al. (2015). Recent studies suggest that foraging behavior of marine invertebrates, such as crustaceans, is potentially altered under high pCO_2_, due to a disturbance of olfactory sensing (Briffa et al., 2012). Altered chemosensory behavior and feeding efficiency upon low-pH exposure have also been observed in crustaceans *P. bernhardus* (de la Haye et al., 2012). Briffa et al. (2012) reviewed recent evidences that exposure to elevated pCO_2_ disrupts the ability to detect prey and food of tropical reef fish and hermit crabs.

In marine crustaceans, behavioral change appears to occur via info-disruption. There are two routes through which exposure to high pCO_2_ could lead to info-disruption. One possibility is that chemoreception, the ability to detect chemical cues, is impaired. Marine animals depend on the detection of chemical cues in order to obtain information about their environment (Hay, 2009) and any change of seawater chemistry could therefore interfere with the detection or recognition of these cues. Besides, elevated pCO_2_ disrupts the ability of some marine invertebrates to maintain their acid–base balance (Pörtner et al., 2004; Spicer et al., 2007), and this physiological mechanism may have downstream effects on behavior. The extension of foraging time under high pCO_2_ may be a more widespread phenomenon. It was also significantly longer in the sea urchin *Strongylocentrotus fragilis* at low pH 7.14 (Barry et al., 2014).

The searching, breaking, eating, and handling times increased when crabs were exposed to elevated pCO_2_ conditions. This was similar to the research in the mud crab *P. herbstii* (Dodd et al., 2015) and the crayfish *Cambarus bartonii* (Allison et al., 1992). Also, acidification reduced the locomotory activity of the hermit crab *P. bernhardus* when either presented with an improved shell choice (de la Haye et al., 2011) or exposed to prey cues (de la Haye et al., 2012), and reduced the swimming ability of a penaeid shrimp (Dissanayake and Ishimatsu, 2011). The associated limitation in performance was possibly mediated through disturbance of acid-base regulation and reduced claw strength. Increases in prey-handling time were largely due to increases in the time taken to glean and ingest flesh, for which time taken to break the shell became important. In addition, the negative effect of CO_2_ on prey handling efficiency appears to have resulted from limited capacity to increase peak MO_2_.

## Conclusion

The effects of elevated pCO_2_ on feeding behavior and energy metabolism of the brown crab *C. pagurus* were tested. At the tested levels, decreased pH had negative effects on the foraging behavior and energy metabolism. Feeding performance and SDA of *C. pagurus* strongly declined with increasing pCO_2_ as a consequence of shifts in prey selection toward smaller mussels, impaired foraging behavior and increased energy demand for maintenance, which left less energy for digestion. Recovery may occur but over extended periods. Impaired foraging efficiency concomitantly with increased energy demand of *C. pagurus* under high pCO_2_ may have negative consequences on their overall performance, possibly also reproduction and survival, potentially affecting the dynamics of the entire population. We conclude that *C. pagurus* in the North Sea may be negatively affected by elevated pCO_2_. Our study also indicates that the overall feeding pressure by crabs on mussels can be expected to decrease with elevated pCO_2_. Since *C. pagurus* is among the most important predators controlling the distribution and abundance of this dominant filter feeder, considerable consequences at ecosystem level can be expected in the future.

## Author Contributions

YW, MH, FW, DS, and H-OP are the guarantors of the integrity of the entire study. YW, DS, and H-OP developed the study concepts. YW designed the study. YW and MH conducted the experiments. YW, MH, FW, and DS analyzed/interpreted the data. YW, MH, DS, and H-OP defined the content of the manuscript. YW and MH prepared a first draft of the manuscript. YW, MH, FW, DS, and H-OP edited and revised the manuscript and approved the final manuscript version.

## Conflict of Interest Statement

The authors declare that the research was conducted in the absence of any commercial or financial relationships that could be construed as a potential conflict of interest.
